# Editorial: Natural bioactives: a promising avenue for metabolic syndrome therapy

**DOI:** 10.3389/fnut.2026.1850579

**Published:** 2026-04-27

**Authors:** Manuela Machado, Irene Gouvinhas, Sara Silva, Eduardo M. Costa

**Affiliations:** 1 CBQF - Centro de Biotecnologia e Química Fina – Laboratório Associado, Escola Superior de Biotecnologia, Universidade Católica Portuguesa, Porto, Portugal; 2 Centre for the Research and Technology of Agro-Environmental and Biological Sciences, University of Trás-os-Montes and Alto Douro, Vila Real, Portugal

**Keywords:** inflammation, metabolic reprogramming, metabolic syndrome, natural bioactives, nutraceuticals

Metabolic syndrome (MetS) now affects approximately one in three adults worldwide—a prevalence that continues to rise alongside global rates of obesity, sedentary behavior, and dietary transition. More than a diagnostic cluster, MetS represents a state of perturbed metabolic signaling: obesity, hypertension, dyslipidaemia, and insulin resistance do not merely co-occur but reinforce one another through shared inflammatory, oxidative, and endocrine mechanisms (1). The consequence is a substantially elevated risk of type 2 diabetes, cardiovascular disease, and end-organ damage (2). Yet conventional pharmacological strategies remain largely reactive, targeting individual risk factors once they emerge rather than correcting the upstream metabolic dysregulation that drives them (3). This gap—between symptom suppression and genuine metabolic restoration—has become one of the defining challenges of modern preventive medicine, and it is precisely this gap that natural bioactive compounds have begun to address.

Natural compounds derived from plants, fungi, marine organisms, and food matrices have long been recognized for their biological activity, but their systematic investigation as metabolic health modulators is a recent development. Phenolics, flavonoids, alkaloids, terpenoids, bioactive peptides, and essential fatty acids have each been shown to engage with key nodes of metabolic regulation—insulin sensitivity, lipid turnover, oxidative balance, and inflammatory cascades. What makes this approach particularly compelling is not the potency of any single molecule, but the breadth of their combined action: unlike most pharmaceuticals, natural bioactives tend to act across multiple pathways simultaneously, a property that aligns well with the multi-factorial character of MetS (4). Nonetheless, the field has struggled with persistent questions regarding compounds bioavailability, their pharmacokinetic predictability, standardization across sources, and the translation of preclinical promise into clinical evidence. This Research Topic was designed to confront those questions directly.

Behind this Research Topic lies a unifying idea: that natural bioactives do not merely ameliorate individual symptoms of MetS but can contribute to the rewiring of the underlying metabolic dysfunction. “Rewiring” is not a word chosen lightly. It implies intervention at the regulatory networks level—gene expression, enzyme activity, receptor sensitivity, cellular energy homeostasis—rather than at the level of isolated biomarkers. The studies gathered here, spanning mechanistic *in vitro* and *in vivo* work, systematic reviews, and clinical meta-analyses, collectively support this framing, while also illuminating where the science remains incomplete.

A first line of evidence is provided by studies examining dietary and plant-derived bioactives as functional modulators of metabolic health. Khormi et al. demonstrated that supplementation with pomegranate peel and Aloe vera gel improved antioxidant status and intestinal integrity—findings that carry a dual significance. Mechanistically, they illustrate how bioactive-rich agro-industrial by-products can be repositioned as therapeutic interventions; conceptually, they reinforce nutrition as an upstream modulator of disease, capable of acting at the level of barrier function and redox homeostasis before systemic metabolic disturbance takes hold. This sustainability-health nexus is, we believe, one of the more underappreciated dimensions of the bioactives field.

At the mechanistic level, Chang et al. examined the effects of α-lipoic acid under stress conditions, demonstrating its capacity to regulate oxidative balance, inflammatory signaling, and apoptosis in hepatic tissue. This contribution speaks directly to the rewiring concept: it shows that a single bioactive compound can act upon multiple control points within the same metabolic pathway, modulating the liver's response to stress rather than simply attenuating one downstream marker. The liver is not incidental here—as the primary site of lipid and glucose metabolism, hepatic dysregulation is both a consequence and a driver of MetS, and the ability of natural compounds to reprogram cellular stress responses in this organ deserves particular attention.

Bridging experimental findings with clinical relevance, Fang et al. provided a systematic review and meta-analysis evaluating the effects of curcumin on lipid profiles and body mass index. By consolidating evidence across trials, this contribution does more than confirm curcumin's metabolic activity—it demonstrates the field's growing maturity. The transition from mechanistic studies to rigorous evidence synthesis is a critical step toward the integration of bioactive-based strategies into clinical practice, and meta-analyses of this kind provides the foundation upon which therapeutic guidelines can in the future be built.

Extending this perspective to organ-level interactions, Lazzarin et al. described the impact of nut consumption in chronic kidney disease. While centered on renal outcomes, this work speaks directly to the bidirectional relationship between kidney function and cardiometabolic risk and that dietary interventions which are effective in one domain are likely to exert effects on other. More broadly, this contribution exemplifies a systems-level view of metabolic health—one in which the therapeutic relevance of a food-based intervention cannot be fully appreciated by examining a single organ or outcome in isolation.

The last two contributions enrich the Research Topic by pointing toward emerging mechanisms and underexplored sources. Sun et al. review on natural compounds involved in the regulation of fatty acid oxidation in diabetic kidney disease, highlighted the potential for metabolic reprogramming, particularly the restoration of mitochondrial β-oxidation capacity, as a critical therapeutic axis. This framing of bioactive compounds actions as reprogramming agents, rather than supplementation, is conceptually significant: it positions these compounds as mediators of metabolic recalibration rather than mere antioxidants or anti-inflammatory molecules. Complementarily, Rampengan et al. highlighted the potential of marine terpenoids in mitigating oxidative stress associated with diabetes, drawing attention to marine ecosystems as a largely underutilized source of structurally diverse bioactive scaffolds. Given the chemical novelty of many marine-derived compounds, this line of research may prove particularly productive for identifying new mechanisms of action.

Taken together, the contributions in this Research Topic converge on a set of shared mechanisms: antioxidant protection, immunomodulation, lipid metabolism regulation and organ-specific functional protection, which collectively overlap into the pathophysiology of MetS with considerable precision. What is striking is not that any single compound addresses all of these axes, but that the accumulated evidence points toward a landscape in which combinations of bioactives, acting across complementary pathways, may ultimately prove more effective than any individual agent. This multi-target capacity is not simply a pharmacological convenience; it reflects a biological logic, insofar as the dysregulation underlying MetS is in itself multi-targeted in nature.

At the same time, the studies in this Research Topic are candid about what remains unresolved. Bioavailability continues to limit the translation of many *in vitro* and animal findings to human contexts. Pharmacokinetic profiles are incompletely characterized for most natural compounds, and the variability introduced by differences in source material, extraction method, and formulation complicates cross-study comparisons. Clinical evidence, while growing, remains heterogeneous in quality and scale. These are not minor caveats, they represent genuine barriers to the therapeutic deployment of natural bioactives and addressing them will require coordinated investment in standardized methodology, delivery system innovation and adequately powered clinical trials.

Looking forward, we believe the most productive direction for the field lies in the integration of systems-level methodologies with precision nutritional frameworks. Omics approaches—genomics, proteomics, metabolomics—offer the resolution needed to characterize individual responses to bioactive interventions and to identify the molecular signatures that predict therapeutic efficacy. Advanced delivery systems, including nanoencapsulation and biopolymer matrices, may substantially enhance bioavailability for compounds currently limited by poor absorption. Last but not least, well-designed clinical studies anchored in mechanistic hypotheses, will be essential to establish the dose-response relationships and patient-specific contexts in which bioactive-based strategies are most likely to succeed.

The concept of rewiring metabolic syndrome thus extends beyond molecular targets. It encompasses how bioactives are sourced, characterized, optimized for delivery and evaluated in populations with the heterogeneity that clinical reality demands. This Research Topic does not resolve all these questions—nor could any single Research Topic —but it advances them, and in doing so contributes to a broader paradigm shift: from the management of MetS as a static constellation of risk factors, toward its active recalibration through integrated, bioactive-driven nutritional strategies. We hope the work gathered here will serve as both a foundation and a provocation for the research that follows.
